# Reverse-transcriptase polymerase chain reaction versus chest computed tomography for detecting early symptoms of COVID-19. A diagnostic accuracy systematic review and meta-analysis

**DOI:** 10.1590/1516-3180.2020.034306072020

**Published:** 2020-08-19

**Authors:** Márcio Luís Duarte, Lucas Ribeiro dos Santos, Andrea Carla de Souza Contenças, Wagner Iared, Maria Stella Peccin, Álvaro Nagib Atallah

**Affiliations:** I MD, MSc. Musculoskeletal Radiologist, WEBIMAGEM, São Paulo (SP), Brazil; Doctoral Student in Evidence-Based Health Program, Universidade Federal de São Paulo (UNIFESP), São Paulo (SP), Brazil.; II MD, MSc. Endocrinologist and Professor of Physiology and Internal Medicine, Centro Universitário Lusíada (UNILUS), Santos (SP), Brazil; Doctoral Student in Evidence-Based Health Program, Universidade Federal de São Paulo (UNIFESP), São Paulo (SP), Brazil.; III MD. Pulmonologist and Professor of Emergency Medicine. Centro Universitário Lusíada (UNILUS), Santos (SP), Brazil; Master’s Degree Student in Evidence-Based Health Program, Universidade Federal de São Paulo (UNIFESP), São Paulo (SP), Brazil.; IV MD, PhD. Supervising Professor of the Postgraduate Evidence-Based Health Program, Universidade Federal de São Paulo (UNIFESP), São Paulo (SP), Brazil.; V PT, PhD. Associate Professor, Department of Human Movement Sciences, and Advisor, Postgraduate Evidence-Based Health Program, Universidade Federal de São Paulo (UNIFESP), São Paulo (SP), Brazil.; VI MD, PhD. Head of Evidence-Based Health Department, Universidade Federal de São Paulo (UNIFESP), São Paulo (SP), Brazil.

**Keywords:** COVID-19 [supplementary concept], Coronavirus infections, Real-time polymerase chain reaction, Polymerase chain reaction, Tomography, Radiology, Ground-glass opacities, CT scan, Accuracy

## Abstract

**BACKGROUND:**

A positive real-time reverse-transcriptase polymerase chain reaction (RT-PCR) for SARS CoV-2, from nasopharyngeal swabs, is the current gold standard diagnostic test for this virus and has sensitivity of 60-70%. Some studies have demonstrated a significant number of false-negative RT-PCR tests while displaying significant tomographic findings, in the early days of symptoms of COVID-19.

**OBJECTIVE:**

To compare accuracy between RT-PCR and computed tomography (CT) for detecting COVID-19 in the first week of its symptoms during the pandemic.

**DESIGN AND SETTING:**

Systematic review of comparative studies of diagnostic accuracy within the Evidence-based Health Program of a federal university in São Paulo (SP), Brazil.

**METHODS:**

A systematic search of the relevant literature was conducted in the PubMed, EMBASE, Cochrane Library, CINAHL and LILACS databases, for articles published up to June 6, 2020, relating to studies evaluating the diagnostic accuracy of RT-PCR and chest CT for COVID-19 diagnoses. The QUADAS 2 tool was used for methodological quality evaluation.

**RESULTS:**

In total, 1204 patients with COVID-19 were evaluated; 1045 had tomographic findings while 755 showed positive RT-PCR for COVID-19. RT-PCR demonstrated 81.4% sensitivity, 100% specificity and 92.3% accuracy. Chest CT demonstrated 95.3% sensitivity, 43.8% specificity and 63.3% accuracy.

**CONCLUSION:**

The high sensitivity and detection rates shown by CT demonstrate that this technique has a high degree of importance in the early stages of the disease. During an outbreak, the higher prevalence of the condition increases the positive predictive value of CT.

**REGISTRATION NUMBER:**

DOI: 10.17605/OSF.IO/UNGHA in the Open Science Framework.

## INTRODUCTION

Since COVID-19 pneumonia emerged in Wuhan, China, there has been a search for knowledge that might prevent or minimize its spread.^1^^,^^2^^,^^3^ In just over three months after its initial breakout, it gained worldwide reach such that it affected more than 2.5 million people, with more than 180,000 deaths in more than 200 countries. COVID-19 is caused by the SARS-CoV-2 virus, a member of the Coronaviridae family.^3^ Its transmission occurs mainly through respiratory droplets.^1^

The clinical spectrum of the disease is variable, and the majority of cases are asymptomatic or oligosymptomatic. The most severe cases, with acute respiratory distress syndrome, commonly affect elderly patients with comorbidities.^3^

A positive real-time reverse transcriptase-polymerase chain reaction (RT-PCR) for SARS-CoV-2, from nasopharyngeal swabs, is the current gold standard diagnostic test. The sensitivity of RT-PCR for SARS-CoV-2 is 50-70%;^4^^,^^5^^,^^6^^,^^7^^,^^8^^,^^9^^,^^10^ around 30-40% of patients with early-stage COVID-19 are false-negative.^4^ An inadequate technique for collecting sampling material or low viral load, limited development of nucleic acid detection technology and variation in the detection rate between different manufacturers may all be determinants for false negative results.^4^^,^^11^

Use of computed tomography (CT) is based on the clinical context and time taken to make the diagnosis, especially in relation to use of RT-PCR and other clinical and laboratory investigations.^4^^,^^12^^,^^13^ CT findings do not alter the diagnosis of COVID-19 in cases in which RT-PCR is positive, but they are useful for grading pulmonary involvement and its evolution.^4^^,^^6^^,^^8^ CT has 56-98% sensitivity,^7^ and according to Ai et al., 25% specificity and 68% accuracy.^14^

Ai et al. found that out of 64 patients with an initially negative RT-PCR test, 15 (23.4%) subsequently had a positive RT-PCR (mean time interval of 5.1 ± 1.5 days); ten of these patients (15.6% of those with initial negative RT-PCR) had typical CT findings at the time of the initial negative RT-PCR.^14^ Fang et al. described a 29.4% rate of abnormal CT in patients with initially negative and subsequently positive RT-PCR.^4^^,^^11^

In the minority of patients with high clinical suspicion in the context of the current pandemic, but with negative initial RT-PCR, the presence of typical CT findings could indicate the possibility of COVID-19 earlier, i.e. before sufficient RT-PCR runs have been done to rule out or confirm the diagnosis.^4^^,^^10^

## OBJECTIVES

To determine the accuracy of RT-PCR and CT over the first seven days of symptoms of COVID-19 and which method is more sensitive for early case detection.

## METHODS

### Study model

The study model followed the guidelines for systematic reviews on diagnostic accuracy studies, i.e. Cochrane Diagnostic Reviewer’s Handbook version 5.1.

### Inclusion criteria

The search was performed in accordance with the Preferred Reporting Items for Systematic Reviews and Meta-Analyses (PRISMA) guidelines. We included comparative studies on diagnostic accuracy among patients who underwent both CT and RT-PCR for making the diagnosis of COVID-19 in the initial days of its evolution, regardless of the severity of the disease. We did not put any restrictions on patient age, origin, language or publication status of the study. There was no exclusion regarding population size or patient age. In the case of missing information, the authors of the study in question were contacted by e-mail.

### Participants

The participants were men and women of all ages with suspected COVID-19 who underwent chest CT and RT-PCR during their first week of symptoms.

### Selection of studies and data extraction

The studies selected were those potentially eligible for inclusion in terms of relevance of the articles or abstracts in indexed journals. Two authors performed independent selections for eligibility. In cases of disagreement, a third author was consulted. Data extraction was performed using a standardized form. The selection process was carried out using the Rayyan platform (https://rayyan.qcri.org).^15^

### Evaluation of methodological quality

The QUADAS 2 tool, which is used to evaluate bias and precision, was used in relation to all the eligible studies.^16^ All analyses and diagrams were completed using RevMan 5.3 and MetaDisc 1.4. The study was approved by our institutional review board, under approval number: 8483190420; date: May 4, 2020. The review was registered in the Open Science Framework database.

### Research methods for selecting studies

A thorough systematic search of the relevant literature was conducted in the PubMed, EMBASE, Cochrane Library, CINAHL and LILACS online scientific publication databases, for original articles published up to June 6, 2020, with no language restrictions. The search used the following Medical Subject Headings (MeSH terms): COVID-19; SARS virus; coronavirus infection; Real-Time Polymerase Chain Reaction; Polymerase Chain Reaction; and Tomography, X-Ray Computed. The reference lists of the studies included and the main reviews on the subject were also evaluated. Manual searches were also carried out in these reference lists. The full search strategy is displayed in Table 1.

**Table 1.** Search strategy according to the corresponding database

Database	Search strategy
Cochrane Library	#1 MeSH descriptor: [SARS Virus] explode all trees #2 MeSH descriptor: [Coronavirus Infections] explode all trees #3 MeSH descriptor: [Real-Time Polymerase Chain Reaction] explode all trees #4 MeSH descriptor: [Polymerase Chain Reaction] explode all trees #5 MeSH descriptor: [Tomography, X-Ray Computed] explode all trees #6: #1 OR #2 AND #3 OR #4 AND #5
MEDLINE	#1: “COVID-19 [Supplementary Concept]”[MeSH] OR (2019 novel coronavirus infection) OR (COVID19) OR (coronavirus disease 2019) OR (coronavirus disease-19) OR (2019-nCoV disease) OR (2019 novel coronavirus disease) OR (2019-nCoV infection) OR “SARS Virus”[MeSH] OR (Severe Acute Respiratory Syndrome Virus) OR (SARS-Related Coronavirus) OR (Coronavirus, SARS-Related) OR (SARS Related Coronavirus) OR (SARS-CoV) OR (Urbani SARS-Associated Coronavirus) OR (Coronavirus, Urbani SARS-Associated) OR (SARS-Associated Coronavirus, Urbani) OR (Urbani SARS Associated Coronavirus) OR (SARS Coronavirus) OR (Coronavirus, SARS) OR (Severe acute respiratory syndrome-related coronavirus) OR (Severe acute respiratory syndrome related coronavirus) OR (SARS-Associated Coronavirus) OR (Coronavirus, SARS-Associated) OR (SARS Associated Coronavirus) OR “Coronavirus Infections”[MeSH] OR (Coronavirus Infection) OR (Infection, Coronavirus) OR (Infections, Coronavirus) OR (Middle East Respiratory Syndrome) OR (MERS (Middle East Respiratory Syndrome)) #2: “Real-Time Polymerase Chain Reaction”[MeSH] OR (Real Time Polymerase Chain Reaction) OR (Real-Time PCR) OR (PCR, Real-Time) OR (PCRs, Real-Time) OR (Real Time PCR) OR (Real-Time PCRs) OR (Kinetic Polymerase Chain Reaction) OR (Quantitative Real-Time Polymerase Chain Reaction) OR (Quantitative Real Time Polymerase Chain Reaction) OR (Quantitative Real-Time PCR) OR (PCR, Quantitative Real-Time) OR (PCRs, Quantitative Real-Time) OR (Quantitative Real Time PCR) OR (Quantitative Real-Time PCRs) OR (Real-Time PCR, Quantitative) OR (Real-Time PCRs, Quantitative) OR “Polymerase Chain Reaction”[MeSH] OR (Polymerase Chain Reactions) OR (Reaction, Polymerase Chain) OR (Reactions, Polymerase Chain) OR (PCR) OR (Inverse PCR) OR (PCR, Inverse) OR (Inverse Polymerase Chain Reaction) OR (Nested Polymerase Chain Reaction) OR (Nested PCR) OR (PCR, Nested) OR (Anchored PCR) OR (PCR, Anchored) OR (Anchored Polymerase Chain Reaction) #3: “Tomography, X-Ray Computed”[MeSH] OR (X-Ray Computed Tomography) OR (Tomography, X-Ray Computerized) OR (Tomography, X Ray Computerized) OR (Computed X Ray Tomography) OR (X-Ray Computer Assisted Tomography) OR (X Ray Computer Assisted Tomography) OR (Tomography, X-Ray Computer Assisted) OR (Tomography, X Ray Computer Assisted) OR (Computerized Tomography, X Ray) OR (Computerized Tomography, X-Ray) OR (X-Ray Computerized Tomography) OR (CT X Ray) OR (CT X Rays) OR (X Ray, CT) OR (X Rays, CT) OR (Tomodensitometry) OR (Tomography, X Ray Computed) OR (X Ray Tomography, Computed) OR (X-Ray Tomography, Computed) OR (Computed X-Ray Tomography) OR (Tomographies, Computed X-Ray) OR (Tomography, Computed X-Ray) OR (Tomography, Xray Computed) OR (Computed Tomography, Xray) OR (Xray Computed Tomography) OR (CAT Scan, X Ray) OR (CAT Scan, X-Ray) OR (CAT Scans, X-Ray) OR (Scan, X-Ray CAT) OR (Scans, X-Ray CAT) OR (X-Ray CAT Scan) OR (X-Ray CAT Scans) OR (Tomography, Transmission Computed) OR (Computed Tomography, Transmission) OR (Transmission Computed Tomography) OR (CT Scan, X-Ray) OR (CT Scan, X Ray) OR (CT Scans, X-Ray) OR (Scan, X-Ray CT) OR (Scans, X-Ray CT) OR (X-Ray CT Scan) OR (X-Ray CT Scans) OR (Computed Tomography, X-Ray) OR (Computed Tomography, X Ray) OR (X Ray Computerized Tomography) OR (Cine-CT) OR (Cine CT) OR (Electron Beam Computed Tomography) OR (Electron Beam Tomography) OR (Beam Tomography, Electron) OR (Tomography, Electron Beam) OR (Tomography, X-Ray Computerized Axial) OR (Tomography, X Ray Computerized Axial) OR (X-Ray Computerized Axial Tomography) OR (X Ray Computerized Axial Tomography) #4: #1 AND #2 AND #3
EMBASE (OvidSP)	#1: ‘covid 19’/exp OR ‘SARS coronavirus’/exp OR ‘Coronavirus infection’/exp #2: ‘pcr assay kit’/exp OR ‘real time polymerase chain reaction’/exp OR ‘polymerase chain reaction’/exp #3: ‘x-ray computed tomography’/exp #4: #1 AND #2 AND #3
LILACS	#1: MH:”SARS Virus” OR (Virus del SRAS) OR (Vírus da SARS) OR (CoV-SARS) OR (CoV-SRAG) OR (Coronavirus Associado a SARS) OR (Coronavirus Relacionado à Síndrome Respiratória Aguda Grave) OR (SARS-CoV) OR (SRAG-CoV) OR (Vírus SARS) OR (Vírus da Pneumonia Asiática) OR (Vírus da Síndrome Respiratória Aguda Grave) OR (Vírus da Síndrome Respiratória Aguda Severa) OR MH:B04.820.504.540.150.113.937$ OR (covid-19) OR (2019 novel coronavirus infection) OR (COVID19) OR (coronavirus disease 2019) OR (coronavirus disease-19) OR (2019-nCoV disease) OR (2019 novel coronavirus disease) OR (2019-nCoV infection) #2: MH:”Real-Time Polymerase Chain Reaction” OR (Reacción en Cadena en Tiempo Real de la Polimerasa) OR (Reação em Cadeia da Polimerase em Tempo Real) OR (Kinetic Polymerase Chain Reaction) OR (PCR, Quantitative Real-Time) OR (PCR, Real-Time) OR (PCRs, Quantitative Real-Time) OR (PCRs, Real-Time) or (Quantitative Real Time PCR) OR (Quantitative Real Time Polymerase Chain Reaction) OR (Quantitative Real-Time PCR) OR (Quantitative Real-Time PCRs) OR (Quantitative Real-Time Polymerase Chain Reaction) OR (Real Time PCR) OR (Real Time Polymerase Chain Reaction) OR (Real-Time PCR) OR (Real-Time PCR, Quantitative) OR (Real-Time PCRs) OR (Real-Time PCRs, Quantitative) OR MH:E05.393.620.500.706$ OR MH:”Polymerase Chain Reaction” OR (Reacción en Cadena de la Polimerasa) OR (Reação em Cadeia da Polimerase) OR (Anchored Polymerase Chain Reaction) OR (Inverse PCR) or (Inverse Polymerase Chain Reaction) OR (Nested PCR) or (Nested Polymerase Chain Reaction) OR (PCR) or (PCR, Anchored) OR (PCR, Inverse) OR (PCR, Nested) OR (Polymerase Chain Reactions) OR (Reaction, Polymerase Chain) OR (Reactions, Polymerase Chain) OR MH:E05.393.620.500$ #3: MH:”Tomography, X-Ray Computed” OR (Tomografía Computarizada por Rayos X) OR (Tomografia Computadorizada por Raios X) OR (Beam Tomography, Electron) or (CAT Scan, X Ray) OR (CAT Scan, X-Ray) OR (CAT Scans, X-Ray) OR (CT Scan, X Ray) OR (CT Scan, X-Ray) OR (CT Scans, X-Ray) OR (CT X Ray) OR (CT X Rays) OR (Cine CT) OR (Cine-CT) OR (Computed Tomography, Transmission) OR (Computed Tomography, X Ray) OR (Computed Tomography, X-Ray) OR (Computed Tomography, Xray) OR (Computed X Ray Tomography) OR (Computed X-Ray Tomography) OR (Computerized Tomography, X Ray) OR (Computerized Tomography, X-Ray) OR (Electron Beam Computed Tomography) OR (Electron Beam Tomography) OR (Scan, X-Ray CAT) OR (Scan, X-Ray CT) OR (Scans, X-Ray CAT) OR (Scans, X-Ray CT) OR (Tomodensitometry) OR (Tomographies, Computed X-Ray) OR (Tomography, Computed X-Ray) OR (Tomography, Electron Beam) OR (Tomography, Transmission Computed) OR (Tomography, X Ray Computed) OR (Tomography, X Ray Computer Assisted) OR (Tomography, X Ray Computerized) OR (Tomography, X Ray Computerized Axial) OR (Tomography, X-Ray Computer Assisted) OR (Tomography, X-Ray Computerized) OR (Tomography, X-Ray Computerized Axial) OR (Tomography, Xray Computed) OR (Transmission Computed Tomography) OR (X Ray Computer Assisted Tomography) OR (X Ray Computerized Axial Tomography) OR (X Ray Computerized Tomography) OR (X Ray Tomography, Computed) OR (X Ray, CT) OR (X Rays, CT) OR (X-Ray CAT Scan) OR (X-Ray CAT Scans) OR (X-Ray CT Scan) OR (X-Ray CT Scans) OR (X-Ray Computed Tomography) OR (X-Ray Computer Assisted Tomography) OR (X-Ray Computerized Axial Tomography) OR (X-Ray Computerized Tomography) OR (X-Ray Tomography, Computed) OR (Xray Computed Tomography) or (mh:E01.370.350.350.810$) OR MH:E01.370.350.600.350.700.810$) OR MH:E01.370.350.700.700.810$ OR MH:E01.370.350.700.810.810$ OR MH:E01.370.350.825.810.810$ #4: #1 AND #2 AND #3
CINAHL	#1: (SARS Virus) or (CoV-SARS) or (CoV-SRAG) or (Coronavirus Associado a SARS) or (Coronavirus Relacionado à Síndrome Respiratória Aguda Grave) or (SARS-CoV) or (SRAG-CoV) or (Vírus SARS) or (Vírus da Pneumonia Asiática) or (Vírus da Síndrome Respiratória Aguda Grave) or (Vírus da Síndrome Respiratória Aguda Severa) OR (COVID-19) OR (2019 novel coronavirus infection) OR (COVID19) OR (coronavirus disease 2019) OR (coronavirus disease-19) OR (2019-nCoV disease) OR (2019 novel coronavirus disease) OR (2019-nCoV infection) #2: (Real-Time Polymerase Chain Reaction) OR (Real Time Polymerase Chain Reaction) OR (Real-Time PCR) OR (PCR, Real-Time) OR (PCRs, Real-Time) OR (Real Time PCR) OR (Real-Time PCRs) OR (Kinetic Polymerase Chain Reaction) OR (Quantitative Real-Time Polymerase Chain Reaction) OR (Quantitative Real Time Polymerase Chain Reaction) OR (Quantitative Real-Time PCR) OR (PCR, Quantitative Real-Time) OR (PCRs, Quantitative Real-Time) OR (Quantitative Real Time PCR) OR (Quantitative Real-Time PCRs) OR (Real-Time PCR, Quantitative) OR (Real-Time PCRs, Quantitative) OR (Polymerase Chain Reaction) OR (Polymerase Chain Reactions) OR (Reaction, Polymerase Chain) OR (Reactions, Polymerase Chain) OR (PCR) OR (Inverse PCR) OR (PCR, Inverse) OR (Inverse Polymerase Chain Reaction) OR (Nested Polymerase Chain Reaction) OR (Nested PCR) OR (PCR, Nested) OR (Anchored PCR) OR (PCR, Anchored) OR (Anchored Polymerase Chain Reaction) #3: (Tomography, X-Ray Computed) OR (X-Ray Computed Tomography) OR (Tomography, X-Ray Computerized) OR (Tomography, X Ray Computerized) OR (Computed X Ray Tomography) OR (X-Ray Computer Assisted Tomography) OR (X Ray Computer Assisted Tomography) OR (Tomography, X-Ray Computer Assisted) OR (Tomography, X Ray Computer Assisted) OR (Computerized Tomography, X Ray) OR (Computerized Tomography, X-Ray) OR (X-Ray Computerized Tomography) OR (CT X Ray) OR (CT X Rays) OR (X Ray, CT) OR (X Rays, CT) OR (Tomodensitometry) OR (Tomography, X Ray Computed) OR (X Ray Tomography, Computed) OR (X-Ray Tomography, Computed) OR (Computed X-Ray Tomography) OR (Tomographies, Computed X-Ray) OR (Tomography, Computed X-Ray) OR (Tomography, Xray Computed) OR (Computed Tomography, Xray) OR (Xray Computed Tomography) OR (CAT Scan, X Ray) OR (CAT Scan, X-Ray) OR (CAT Scans, X-Ray) OR (Scan, X-Ray CAT) OR (Scans, X-Ray CAT) OR (X-Ray CAT Scan) OR (X-Ray CAT Scans) OR (Tomography, Transmission Computed) OR (Computed Tomography, Transmission) OR (Transmission Computed Tomography) OR (CT Scan, X-Ray) OR (CT Scan, X Ray) OR (CT Scans, X-Ray) OR (Scan, X-Ray CT) OR (Scans, X-Ray CT) OR (X-Ray CT Scan) OR (X-Ray CT Scans) OR (Computed Tomography, X-Ray) OR (Computed Tomography, X Ray) OR (X Ray Computerized Tomography) OR (Cine-CT) OR (Cine CT) OR (Electron Beam Computed Tomography) OR (Electron Beam Tomography) OR (Beam Tomography, Electron) OR (Tomography, Electron Beam) OR (Tomography, X-Ray Computerized Axial) Ography, X Ray Computerized Axial) OR (X-Ray Computerized Axial Tomography) OR (X Ray Computerized Axial Tomography) #4: #1 AND #2 AND #3

## RESULTS

### Studies selected

**Figure 1.**
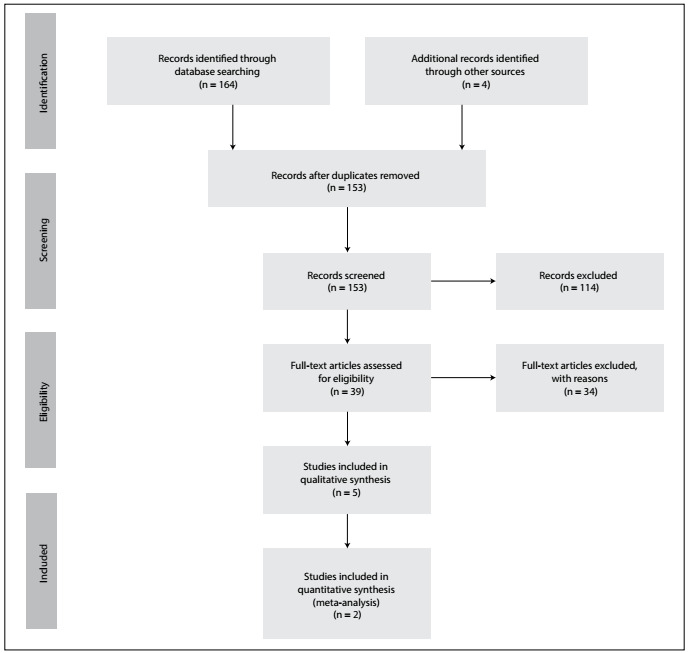
PRISMA 2009 flow diagram.

The systematic review yielded 168 studies. At the end of the analysis, five studies. ^9^^,^^11^^,^^14^^,^^17^^,^^18^ were deemed to meet the inclusion criteria and presented acceptable quality according to the QUADAS 2 tool. These studies were thus included in the systematic review (Figure 1). Among these, two studies were included in the meta-analysis.^9^^,^^17^

**Figure 2.**
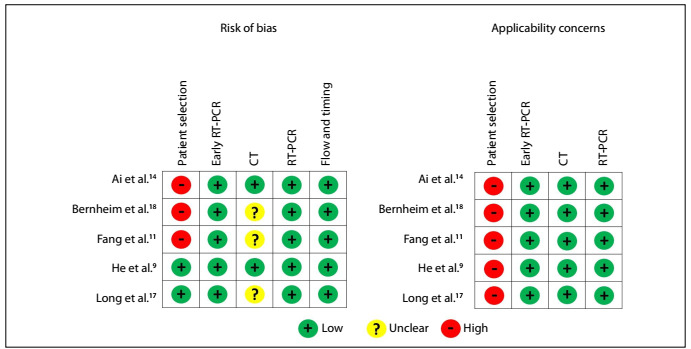
QUADAS 2 risk of bias and applicability concerns.

In all the studies, there was high concern about applicability. Moreover, in three of the five studies, a high risk of bias was also perceived. It was not clear in most studies whether the radiologist who reported the CT scan had access to the RT-PCR results (Figure 2).

### Analysis on the studies

Table 2 provides a summary of the findings from the main studies included. In total, 1204 patients with COVID-19 that were evaluated. Among these, 1045 had tomographic findings (detection rate of 86.7%) and 755 showed positive RT-PCR for COVID-19 (detection rate of 62.7%), with a significant difference in detection rate of 24.0%.

**Table 2.** Summary of study findings

Study	Total number of COVID-19 patients included	Positive early RT-PCR	Positive early CT	Comments
Ai 2020^14^	1014	601	888	Average interval between tests: 1 day (less than or equal to 7 days). 308 patients had negative RT-PCR and positive CT. 21 patients had positive RT-PCR and negative CT. 580 patients with positive RT-PCR had positive CT. 258 multiple RT-PCR: average conversion time was 5 days. 10 out of 15 RT-PCR conversions had CT findings when the RT-PCR was negative.
Bernheim 2020^18^	121	61 out of 69 tested in the early and intermediate group	46 out of 69 tested in the early and intermediate group	121 patients divided in three groups: Early group: 0-2 days of symptoms. Intermediate group: 3-5 days of symptoms. Late group: 6-12 days of symptoms.
Fang 2020^11^	51	36	50	Average time between symptoms and CT or RT-PCR: 3 days
He 2020^9^	34	27	26	Performed both tests in the first 2-5 days of symptoms. 48 initial RT-PCR were true negative. 7 initial RT-PCR were false negative. 46 CT were true negative. 8 CT were false positive. 2 CT were false negative.
Long 2020^17^	36	30	35	Performed both tests during the initial presentation of the disease. 30 patients had positive RT-PCR and 35 had positive CT. 6 patients had negative RT-PCR and positive CT. 1 patient had negative CT and positive RT-PCR. 51 patients had negative RT-PCR and positive CT.

Regarding tomographic pattern changes, Ai et al.^14^ found that out of their 888 patients with positive CT results, 409 showed ground-glass opacities and 447 had consolidations; 801 had bilateral findings. Also, 42% of the patients showed improvement on CT before the RT-PCR became negative; and 3.5% showed worsened CT with negative RT-PCR. The CT sensitivity was 96.5% and its specificity was 25.4%; the positive predictive value was 65.3% and the negative predictive value was 83.3%. The first RT-PCR performed on the patients presented sensitivity of 59.2%.

Bernheim et al. evaluated patients divided into three groups concerning the onset of symptoms: early (0-2 days), intermediate (3-5 days) and late (6-12 days). They found that 56% (20 patients out of 36) had an absence of ground-glass opacity and consolidation in the first two days, while 9.0% (three patients out of 33) showed this in the intermediate group and 4% (one patient out of 25) in the late group. RT-PCR was positive in 91.6% of the patients in the early group (33 patients out of 36); 84.4% in the intermediate group (28 patients out of 33); and 92.0% in the late group (23 patients out of 25). One patient with absence of ground-glass opacity and consolidation in the early group showed negative RT-PCR findings. RT-PCR presented sensitivity of 88.4% and CT of 66.6% over the first five days of symptoms.

In the study by Fang et al.,^11^ study, 36 CT-positive cases showed typical changes: sparse, subpleural and peripheral ground-glass opacities, commonly in the lower lobes. The CT sensitivity was 98.0%. The first RT-PCR performed on the patients presented sensitivity of 70.5%.

He et al.^9^ compared use of CT and RT-PCR among 82 patients with suspected pneumonia, including COVID-19 pneumonia. The two experienced radiologists who evaluated all chest CT scans demonstrated good interobserver agreement. All the patients underwent chest CT and initial RT-PCR on the same day. The 34 COVID-19 patients had confirmation through RT-PCR, but not necessarily from the initial RT-PCR. The initial RT-PCR had 79% sensitivity, 100% specificity and 92% accuracy. The chest CT had 77% sensitivity, 96% specificity and 88% accuracy. He et al. also analyzed the two tests used in conjunction, and concluded that jointly they presented 88% sensitivity, 100% specificity and 98% accuracy.^9^ In the study by He et al.,^9^ eight patients with tomographic changes had pneumonia other than COVID-19. It was possible to calculate the positive predictive value of CT, which was 85.1%.

Long et al.^17^ also compared the tomographic findings of patients with COVID-19 pneumonia and non-COVID-19 pneumonia. The upper lobes of the lungs were more affected on CT in COVID-19 cases (right: 52.7% versus 37.3%; left: 55.6% versus 33.3%); the other lobes did not show any significant difference. There was also a difference in peripheral involvement, which was more common in cases of pneumonia caused by COVID-19. The sensitivity of CT was 97.2%. The first RT-PCR performed on the patients presented sensitivity of 84.6% and the negative predictive value was 89.4%. In the study by Long et al.,^17^ 51 patients with tomographic findings had pneumonia other than COVID-19. This makes it possible to infer that CT presents high specificity. The positive predictive value for CT was calculated as 58.6%.

### Accuracy assessment

**Figure 3.**
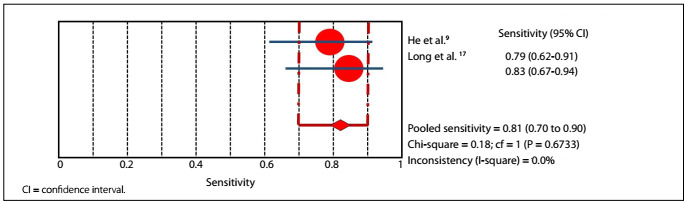
Sensitivity graph: RT-PCR.

**Figure 4.**
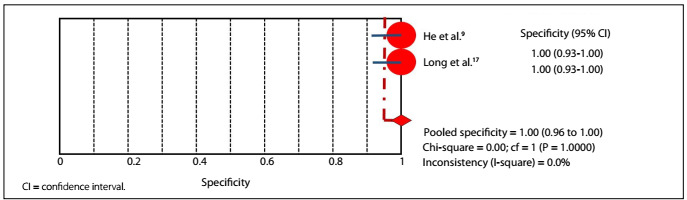
Specificity graph: RT-PCR.

**Figure 5.**
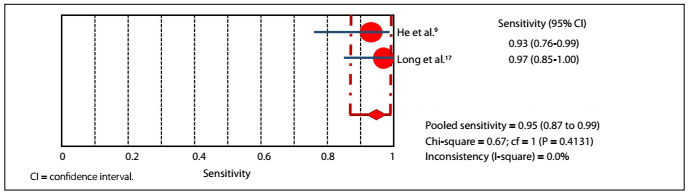
Sensitivity graph: chest CT.

**Figure 6.**
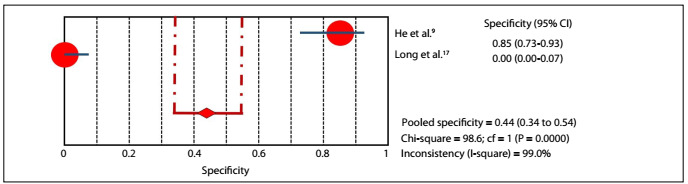
Specificity graph: chest CT.

In the accuracy evaluations of the studies by He and Long,^9^^,^^17^ RT-PCR demonstrated 81.4% sensitivity (95% confidential interval: 70.3-89.7%) and 100% specificity (95% confidential interval: 96.3-100%), with P-value lower than 0.05, and 92.3% accuracy (Figures 3**and**4). In the same studies, CT demonstrated 95.3% sensitivity (95% confidential interval: 86.9-99.0%) and 43.8% specificity (95% confidential interval: 34.1-53.8%), with P-value lower than 0.05, and 63.3% accuracy (Figures 5**and**6).

All the data in these five studies were retrospective and were obtained during the epidemic period in the regions where these studies were conducted.

## DISCUSSION

The symptoms of COVID-19 consist mainly of fever, fatigue and dry cough, with gradual dyspnea in some cases, and acute respiratory distress syndrome and multiple organ dysfunction in severe cases requiring intensive treatment.^1^^,^^2^^,^^19^^,^^20^ While the majority of patients, about 80%, have mild symptoms; older patients, especially those above 70 years old and those with underlying conditions, such as cardiovascular disease, diabetes, chronic respiratory diseases and oncological diseases, have a higher mortality rate of up to 15%.^3^

In addition to the most common pattern of peripheral and bilateral ground-glass injuries, other patterns of lung injury may be observed.^6^^,^^7^^,^^20^^,^^21^^,^^22^^,^^23^^,^^24^^,^^25^ Pulmonary consolidations are present in 2-64% of the cases and form an indicator of disease progression, thus serving as a warning sign for the severity of the patient’s condition. Reticular pattern lesions have lower incidence than consolidations and opacities.^6^^,^^26^

The crazy-paving pattern is present in about 5-36% of the cases, while bronchial wall thickening is present in 10-20%.^6^^,^^27^ Pleural changes are present in 32%, with pleural thickening; however, pleural effusion occurs in only 5% of the cases.^6^^,^^24^ Pulmonary fibrosis occurs in 17% of the cases and pulmonary nodules smaller than three centimeters in size, in 3-13%.^6^ The incidence of lymph node enlargement is about 4-8% and pericardial effusion occurs in approximately 5%. The latter is an indicator of severity.^6^^,^^19^ Vascular thickening is characterized in 59% of the cases.^7^ The radiological findings tend to become worse seven days after the onset of symptoms and show improvement 14 days after the onset of symptoms.^3^

In the current pandemic situation, despite the low specificity of CT (25%), this technique can be used to isolate patients and institute treatment at an early stage, since it presents sensitivity of about 88.9%, starting from the early day of symptoms.^4^^,^^10^^,^^20^^,^^28^ In comparison with this, chest X-ray shows abnormalities in 59.1% of the cases and in 76.7% among serious cases.^4^^,^^23^

Xie et al.^29^ reported on a case series in which they performed RT-PCR and CT on the same day, regardless of the duration of the patients’ symptoms. They found that out of their 167 patients, 162 were positive according to RT-PCR and 160 were positive according to CT. Seven patients were positive on RT-PCR and negative on CT; and five patients were positive on CT and negative on RT-PCR. CT presented 95.0% of sensitivity, while RT-PCR presented 97.0%. Concerning false-negative data, CT showed 4.0%.

Barbosa et al. evaluated 91 patients with suspected pneumonia until 30 days after their initial symptoms and performed RT-PCR and chest CT on the same day. Sixty-three of their patients had symptoms for seven days or less and, among these patients, two were positive on RT-PCR and negative on CT, with a CT false-negative rate of 3.1%. For 28 patients, both tests were negative; and for 15, both tests were positive.^10^

It should, however, be noted that a normal CT scan cannot be used to rule out a diagnosis of COVID-19,^21^^,^^26^^,^^30^ although there is some evidence to suggest that the negative predictive value of CT is greater for symptoms lasting longer than one week.^4^ It also needs to be taken into account that CT may be normal in cases with positive RT-PCR in 2-20% cases, according to studies by Yang et al., Guan et al. and Chung et al.^4^^,^^19^^,^^23^^,^^31^

Moreover, 54-70.8% of asymptomatic people who have had contact with symptomatic patients and who are COVID-19-positive according to RT-PCR may present a change on CT.^4^^,^^5^^,^^32^ Long et al. analyzed 37 asymptomatic individuals, who had come into contact with RT-PCR-confirmed patients, and reported that 21 (56.7%) of these individuals had positive CT findings.^33^ Inui et al.^5^ detected pulmonary opacity on CT in 24 out of 30 symptomatic patients (80%); however, in 82 asymptomatic patients, 44 (54%) had opacities on CT.^5^ Shi et al. also reported occurrences of CT abnormalities in asymptomatic patients.^24^ Furthermore, in symptomatic cases, they found greater extent of the lesion, along with areas of consolidation predominating over ground-glass opacities.^5^^,^^24^

Bai et al. assessed the performance of radiologists in differentiating CT results between those from patients with COVID-19 pneumonia and those from patients with non-COVID-19 pneumonia.^7^ These radiologists achieved accuracy ranging from 72 to 97%, with sensitivity of 70-94% and greatly varying specificity (24-94%).^7^

Therefore, the role of CT in confirmed cases of COVID-19 after the results from RT-PCR have been obtained is the same as in relation to any other viral infection, in that it can be used to do the following:^4^

- Add diagnostic value for patients with pre-existing lung diseases.
- Help diagnose complications or investigate a clinically discordant condition: positive to negative turnover RT-PCR, but increased hypoxia.
- Find coexisting or underlying diagnoses.

Although CT is very sensitive at the onset of symptoms, in comparison with RT-PCR, it still may not reveal the characteristic pattern of COVID-19 in all cases. Hence, it remains difficult to differentiate COVID-19 from other viral causes of pneumonia.^7^ According to Bai et al., although making the diagnosis of pneumonia due to COVID-19 is possible via CT, subtle or atypical presentations can lead to a wrong diagnosis.^7^

Our findings showed that CT outperformed RT-PCR in making an early diagnosis of COVID-19 in suspected cases. Both from previous findings and ours, we suggest that an early evaluation protocol should include applying CT when RT-PCR is negative. This could guide clinicians’ treatment and patient isolation criteria, in order to avoid virus dissemination. Our meta-analysis showed that CT had specificity of 43.8% and sensitivity of 95.3%, and both of these values are higher than those in the recent literature.

All the studies evaluated were conducted among in patients with COVID-19 that confirmed within the first seven days of symptoms by means of RT-PCR. However, this test was not necessarily the first to be performed on suspected patients, within the epidemic period in the country in which these tests were performed. Therefore, the positive predictive value and detection rate of CT findings in patients with COVID-19 will be higher than it would be outside the epidemic period.

Although RT-PCR is the gold standard for diagnosing COVID-19, it presents a significant percentage of false-negative tests in the early days of symptoms of the disease (0-7 days). On the other hand, even though CT is a test with presumably low specificity,^26^ thereby allowing several differential diagnoses,^8^ it detects patterns compatible with COVID-19. It has presented very high sensitivity and significant positive predictive value and detection rate in the epidemic period.^8^^,^^26^

## CONCLUSION

The high sensitivity and detection rate of CT demonstrate that it has a high degree of importance in the early stages of the disease, even greater than RT-PCR. During an outbreak, the higher prevalence of the condition raises the positive predictive value of CT. However, the low specificity of CT (43.8%) also needs to be considered. Outside of pandemic times, its positive predictive value for this condition should decrease proportionally with the decline in the prevalence of the disease in the population.
